# Rapid diagnosis of *Helicobacter pylori* infection status based on endoscopic features and deep learning algorithms

**DOI:** 10.3389/fpubh.2026.1765267

**Published:** 2026-02-04

**Authors:** Xinying Yu, Lianyu Li, Qiang He

**Affiliations:** 1 Department of Gastroenterology, Beijing Tiantan Hospital, Capital Medical University, Beijing, China; 2 Department of Electronic Information and Communication, Huazhong University of Science and Technology, Wuhan, Hubei, China

**Keywords:** AI-assisted diagnosis, classification model, endoscopic diagnosis, *Helicobacter pylori*, infection status

## Abstract

**Background and aims:**

Endoscopic visualization for the diagnosis of *Helicobacter pylori* (HP) infection status is highly important for helping endoscopists quickly understand the status of gastric background mucosa and assisting in subsequent diagnosis and treatment. In this study, a deep learning algorithm was designed to construct a three-class classification model of HP infection status, providing a new approach to address the subjectivity in the interpretation of endoscopic features.

**Methods:**

The clinical data of patients who completed gastroscopy were collected, and 16 endoscopic features were evaluated and recorded. On the basis of the status of HP infection, the patients were classified into three groups: the current HP infection group (CI), the previous HP infection group (PI), and the negative HP infection group (NI), with 1,000 patients screened in each group. In this study, an HP infection classification model based on the transformer network was constructed, which uses a self-attention mechanism to capture feature associations for the task of HP infection classification and recognition. Model interpretability was achieved by screening key features through SHapley Additive exPlanations (SHAP) value analysis.

**Results:**

A total of 3,000 subjects were included in the study, and comparative analysis revealed that the 1D-transformer model demonstrated superior performance in the HP recognition task. The accuracy, specificity, sensitivity, and F1_scores produced by the model were 98.9 ± 0.28, 98.8 ± 0.14, 99.4 ± 0.27, and 98.9 ± 0.28, respectively. In addition, it has better performance than other algorithms and models. In terms of model interpretability, this study highlights the importance rankings of different features in model decision-making and the directions of their influence. The results show that map redness (SHAP value of 0.220), xanxoma (SHAP value of 0.101), atrophy (SHAP value of 0.065), and intestinal metaplasia (SHAP value of 0.008) are key features for identifying the PI. Diffuse redness (SHAP 0.186), thickened folds (SHAP 0.126), mucus coverage (SHAP 0.094), and nodular changes (SHAP 0.043) are key features for identifying CI. The presence of RAC (SHAP 0.262) and ridge redness (SHAP 0.026) are key features for identifying NI.

**Conclusion:**

This study applies a 1D-transformer model to the task of classifying HP infection status, and compared with other models, it can precisely screen out populations with three different HP infection statuses, with higher performance and reliability.

## Introduction

*Helicobacter pylori* (HP) infection has become a major public health challenge (1, 2). It is not only a major cause of chronic active gastritis but also an independent risk factor for peptic ulcers. More importantly, HP is classified as a Group 1 carcinogen by the WHO, and persistent infection can gradually induce gastric cancer through the pathological process of “chronic superficial gastritis–atrophic gastritis–intestinal metaplasia–dysplasia–gastric cancer” (3, 4). Therefore, achieving early and precise diagnosis of HP infection is not only a core step in controlling the spread of infection and improving the clinical prognosis of patients but also an important strategic measure to reduce the incidence and mortality of gastric cancer (5–7). Although the clinical hazards of HP infection are clear, the current commonly used detection methods still have many limitations, and it is difficult to meet the needs of large-scale screening and precise diagnosis (8, 9).

To overcome the bottleneck of traditional detection techniques, visual diagnosis based on endoscopic features has become a research hotspot in the field of HP infection diagnosis in recent years. Scoring systems such as the Kyoto Gastritis Classification and OLGA/OLGIM staging have been established internationally. However, the interpretation of endoscopic features is highly dependent on the clinical experience and subjective judgment of physicians, and the diagnostic consistency among different levels of hospitals and physicians of different levels of seniority is poor (the kappa value is only 0.4–0.6), which is prone to missed diagnosis and misdiagnosis and limits further improvement of its diagnostic efficacy (5).

In recent years, with the breakthrough progress of artificial intelligence (AI) technology in the field of medical imaging, computer-aided diagnosis (CAD) systems with deep learning as the core have provided a new approach to solve the subjectivity problem of endoscopy feature interpretation. With their powerful feature extraction and pattern recognition capabilities, deep learning algorithms can learn from massive amounts of labeled data and establish standardized diagnostic models. Research has confirmed that AI systems perform significantly better than traditional manual scoring in the field of HP infection diagnosis (10–12).

Current research on AI-assisted HP infection diagnosis focuses mainly on the binary classification differentiation between “current infection” and “no infection” (13, 14). This study breaks through the traditional binary classification diagnostic framework for the first time and presents a three-dimensional typing AI diagnostic model for HP infection of “previous infection/current infection/no infection”. In this study, by integrating endoscopic features and clinical follow-up data, a labeled dataset containing three types of infection states is constructed. The establishment of this three-dimensional typing model not only fills the clinical gap in AI-assisted diagnosis in previously infected populations, increasing AI-assisted diagnosis from the basic screening level of “distinguishing infection or not” to the clinical decision support level of “precisely defining the stage of infection” but also provides a more targeted diagnosis and treatment basis for clinical practice. Therefore, the main contributions of this paper are summarized as follows: (1) For the first time, we adopt deep learning methods to handle the classification task of diagnosing three different infection states of HP and construct a unified diagnostic model that can represent “past infection–current infection–no infection,” achieving a breakthrough from binary classification to multistage infection recognition. (2) Under uniform experimental conditions, a systematic comparison is made between five types of deep learning (transformer, LightResNet, RNN, LSTM, and GRU) models and three traditional machine learning (SVM, XGBoost, and MLP) algorithms. (3) Based on the SHapley Additive exPlanations (SHAP) method, an interpretability analysis of the decision-making process of the HP infection classification model is conducted, revealing the key basis for the model to distinguish different infection stages from the feature contribution level and identifying the core features that have a significant impact on the diagnostic results. These findings support the clinical credibility and generalizability of the model.

## Methods

### Case data

Clinical data for patients who underwent gastroscopy and *HP* testing at Beijing Tiantan Hospital, Capital Medical University, from January 2024 to June 2025 were collected and retrospectively analyzed. The enrolled patients were consecutive patients. The inclusion criteria included an age greater than 18 years, a completed gastroscopy and a diagnosis of chronic gastritis, a biopsy of the gastric mucosa and a complete pathological HP test, and a complete collection of clinical data such as a previous HP eradication history. The exclusion criteria included gastric space-occupying lesions such as gastric cancer, lymphoma, and familial adenomatous polyposis and a previous history of gastric surgery.

### Definitions of observation indicators and data collection

The basic information of all the enrolled patients, including age, sex, past medical history, history of HP infection and treatment status, etc., was collected. Gastroscopy was performed by specialist endoscopists with at least 5 years of endoscopy experience for all the patients. During gastroscopy, white light was used for observation and assessment, and endoscopic photographs before and after irrigation were taken. Two expert endoscopists with more than 5 years of endoscopy experience evaluated all the endoscopy images and assessed and recorded the characteristics under endoscopy, including atrophy, intestinal metaplasia, nodular changes, thickened folds, mucus attachment, diffuse redness, patchy redness, punctate redness, xanthophyloma, hyperplastic polyps, chicken skin-like changes, map-like redness, gastric fundus polyps, multiple white flat elevations, ridge redness, and the presence of a regular arrangement of collecting venules (RAC), yielding a total of 16 features. The numbers and endoscopic manifestations of the 16 features are described in Table 1. A biopsy of the gastric mucosa was subsequently performed on the antrum of the stomach or the lesser curvature of the stomach body.

**Table 1:** Description of the endoscopic features of the enrolled patients.

Feature number	Feature name	Feature description
0	Shrinking	The color of the gastric mucosa fades to pale or grayish-white, the mucosal vascular network is clearly visible, and the mucosa itself becomes thin.
1	Metaplasia of the intestinal skin	The mucosa shows grayish-white, slightly raised patches with a villous or fine granular surface.
2	Nodular changes	The surface of the gastric mucosa is covered with countless uniform, fine nodular protrusions, resembling a cobblestone pavement.
3	Thickened folds	The folds of the stomach wall are abnormally thickened and widened and do not flatten even when inflated.
4	Mucus attachment	The surface of the gastric mucosa is covered with large amounts of white, turbid, reflective lakes or spots of mucus that are not easily washed away by water.
5	Diffuse redness	Extensive, uniform and large red areas appear on the gastric mucosa with unclear boundaries.
6	Patchy redness	Well-defined, irregularly shaped red patches appear on the mucosa, scattered or densely distributed.
7	Punctate redness	Many pinhead-sized red dots are visible on the mucosa, resembling rashes.
8	Xanthophyloma	It presents as a single or multiple slightly raised, flat, yellow or yellowish-white patch.
9	Hyperplastic polyps	Hemispherical or spherical protrusions with clear boundaries that have smooth or slightly rough surfaces are present. Sometimes erosions, congestion or small depressions can be seen at the top, and the surface of some polyps may be lobulated.
10	Chicken skin-like changes	The mucosa of the antrum is diffusely covered with dense clusters of white dots, resembling chicken skin.
11	Map-like redness	The mucosa shows irregularly shaped, meandering red areas that resemble national lines on a map.
12	Gastric fundus gland polyps	Multiple transparent, smooth, hemispherical polyps appear in the fundus or body of the stomach, with the same color as the surrounding mucosa.
13	Multiple white, flat protrusions	Multiple slightly raised, flat white or milky white lesions can be seen on the mucosa of the fundus or body of the stomach.
14	RAC is present	In the lower part of the body of the stomach or in the antrum of the stomach, collecting veins can be seen in a regular reticular or star-shaped distribution, which is a manifestation of normal mucosa.
15	Ridge redness	There are cord-like, linear red streaks on the gastric mucosa, often along the edges of the folds.

### Process and grouping of HP detection

Endoscopic biopsy gastric mucosal tissue was fixed with 10% neutral formalin, embedded in paraffin, sectioned consecutively, and then subjected to Warthin–Starry silver staining. After staining was completed, the surface of the gastric mucosal epithelial cells and the conditions in the glandular fossa were observed under an optical microscope. If gram-negative bacilli were curved or spiral-shaped and rounded at both ends, positive HP infection could be detected and included in the current HP infection group (CI). The previous HP infection group (PI) needs to meet the following two criteria simultaneously: no such characteristic bacteria were observed in this examination, and the patient has a complete record of HP cure. The HP-negative group (NI) is defined as having a negative histopathological result in this examination and no previous history of *H. pylori* infection or treatment.

### Data preprocessing

In the initial processing stage, we first classified all the HP data into three types: PI, CI, and NI, with each sample containing 16 feature points. To eliminate the dimensional differences between features and improve the training effectiveness of the subsequent model, each feature was standardized independently within the training set, validation set, and test set, such that its mean was adjusted to 0 and its variance to 1, ensuring that all features were on the same scale. To address the problem of non-uniform sample sizes across groups, outliers in each class were removed using the three-sigma rule, and then 1,000 samples were randomly selected from each class. All preprocessing steps were applied independently within the training set, validation set, and test set, ensuring class balance and preventing any information leakage between datasets.

### Model architecture

**Figure 1:**
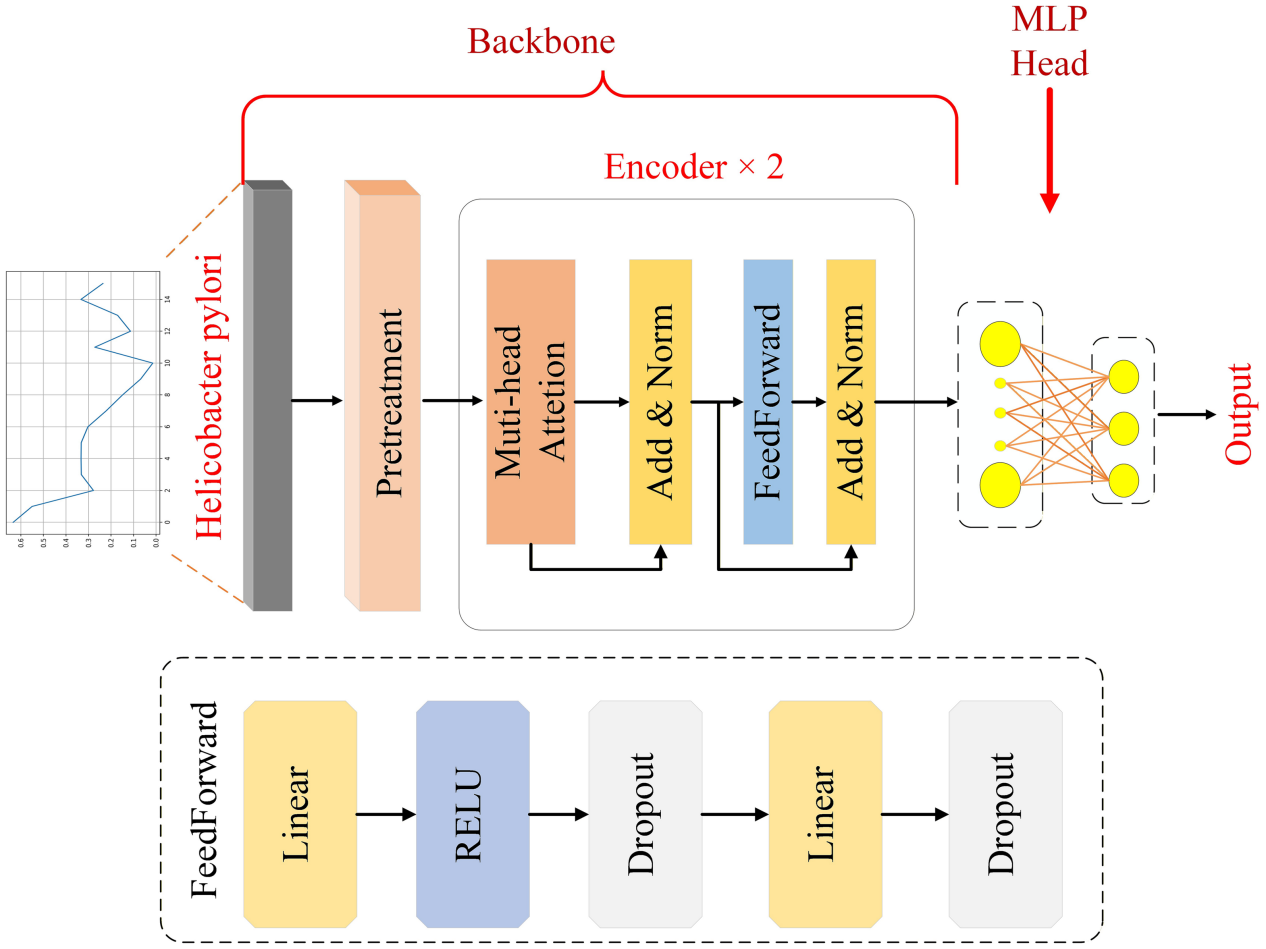
Architecture diagram of the 1D-transformer network model.

This paper presents a 1D-transformer network model for identifying different HP infection status classification tasks in PI, CI, and NI. The network structure, as shown in Figure 1, is divided into two parts: the encoder, and the classification head.

In this study, the collected HP tissue signals are preprocessed first to obtain feature vectors with dimensions of 1 × 16. Given that the 16 input variables are manually defined endoscopic features and do not exhibit an inherent sequential or positional order, an explicit positional encoding layer is not incorporated into the proposed model. Instead, the self-attention mechanism is employed to model inter-feature correlations and relative importance in a permutation-invariant manner.

In the encoding and modeling phase, the module adopts a hierarchical design: the first submodule integrates the multihead attention mechanism and the addition and normalization functions, and the second submodule contains the feedforward neural network, addition and normalization functions, with two encoder layers stacked in total. With this structure, the model is able to extract and reinforce the global and local dependencies of the input features layer by layer. Ultimately, the encoder output is passed to the classification head, which consists of two fully connected layers, enabling three-class prediction of the sample.

### Model evaluation methods

To evaluate the generalization ability of the model in the HP diagnosis task, we first randomly selected one-tenth of the samples from each category (PI, CI, and NI), resulting in a fixed test set of 300 samples. The remaining samples were then divided into 10 subsets for cross-validation. In each round of training, eight subsets were used for training and two subsets were used for validation. This ten-fold cross-validation procedure was repeated 10 times, yielding 10 trained models. Each model was subsequently evaluated on the fixed test set to avoid data leakage, and the final performance of the model was obtained by averaging the evaluation metrics across all 10 models.

To evaluate the model’s performance across different classification tasks, we adopted confusion-matrix–based evaluation criteria. The classification results were obtained by comparing the predicted labels with the corresponding ground-truth categories, from which true positive, true negative, false positive, and false negative outcomes were derived. Based on these outcomes, the performance of the classification network was evaluated using accuracy, specificity, sensitivity, and F1_scores (15).

### Model training

To complete the final classification, we use softmax to calculate the probability of the output. The learning rate is set to 0.0005, the batch size is 16, and the training period is 10. We chose the AdamW optimization algorithm with parameters beta_1 = 0.9, beta_2 = 0.999, and weight decay set to 0.0001. In addition, the cosine annealing strategy is used to dynamically adjust the learning rate so that it gradually decreases during training and becomes smoother in the later stages. The minimum learning rate is set to 0, the preheating phase linearly increases, the number of preheating iterations is 5, the initial learning rate ratio is 0.0002, and the preheating process is carried out in epochs. All the experiments were conducted in an environment equipped with an NVIDIA GeForce RTX 2080 Ti, using the PyTorch deep learning framework.

### Model visualization

To more clearly explain the extent to which different features contribute to model decisions, we introduced SHapley Additive exPlanations (SHAP) values for model interpretability analysis. SHAP is a game theory-based method for explaining the importance of features, which quantifies the impact of each feature on the model output by calculating its marginal contribution across different predictions.

In this study, we used the DeepExplainer method, which is suitable for deep learning models, to interpret the 1D-transformer model. DeepExplainer is capable of combining the structural information within the model to effectively capture the interaction relationships between nonlinear features and is applicable to network architectures with complex attention mechanisms. First, DeepExplainer, which is suitable for deep models, is selected as the interpreter; next, representative samples are selected from the training set to construct the background set; then, the test samples are input, and the SHAP value for each feature is calculated to measure its positive or negative impact on the model output. Finally, the importance and contribution directions of the features are presented through visualizations such as a summary plot to enhance the transparency and comprehensibility of the model. Through SHAP value analysis, we can not only identify the key features that have the greatest impact on the classification results but also reveal the differences in feature contributions when the model predicts different categories, such as the PI, CI, and NI.

## Results

### Patient information

**Figure 2:**
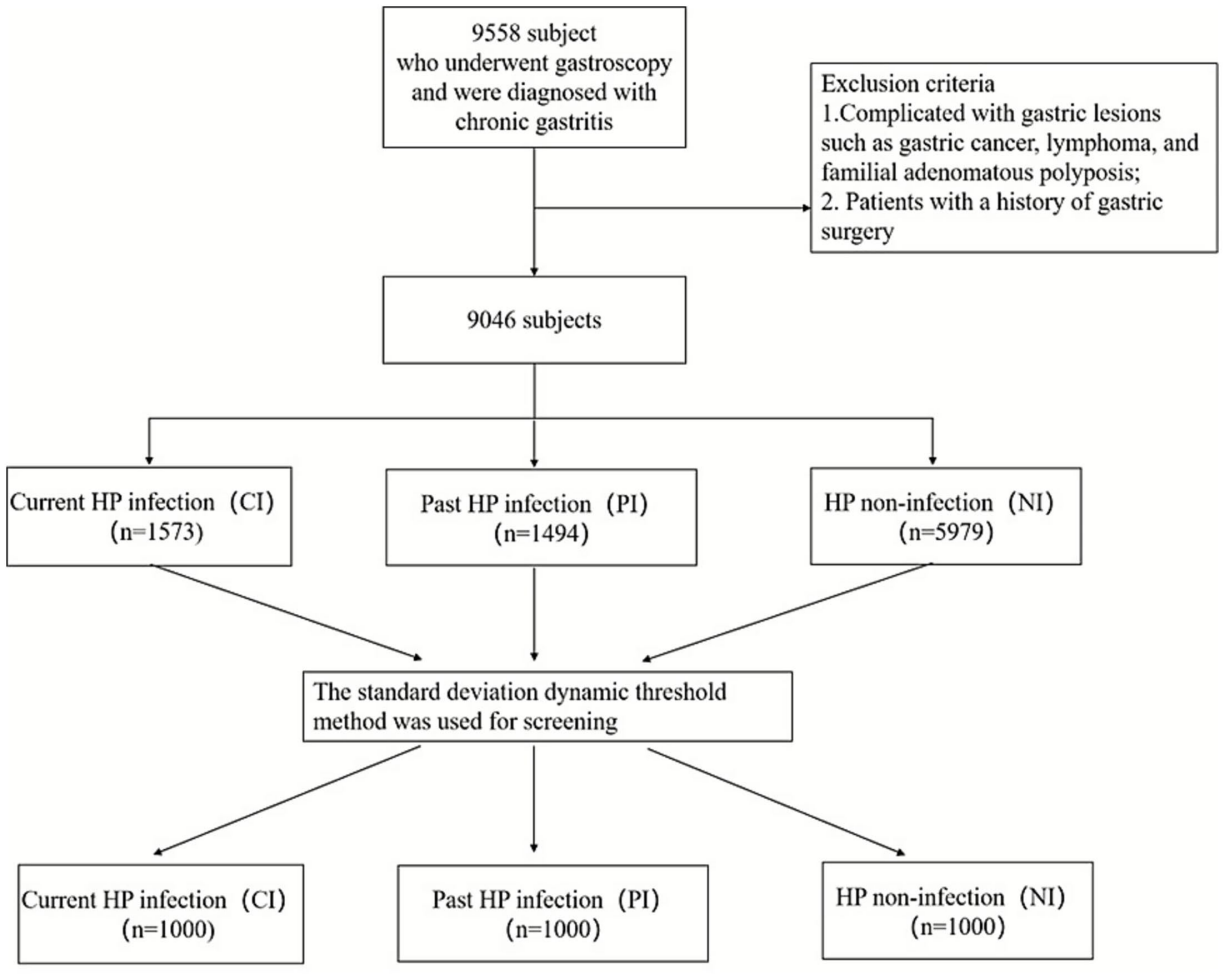
Study population.

A total of 9,046 eligible subjects were included in the study, including 1,573 with current HP infection, 1,494 with previous HP infection, and 5,979 without HP infection. After the data were screened, a total of 3,000 subjects were enrolled, with 1,000 subjects in each of the PI, CI and NI groups. The flowchart of the study is shown in Figure 2.

### Model test results and analysis

#### 1D-transformer

**Figure 3:**
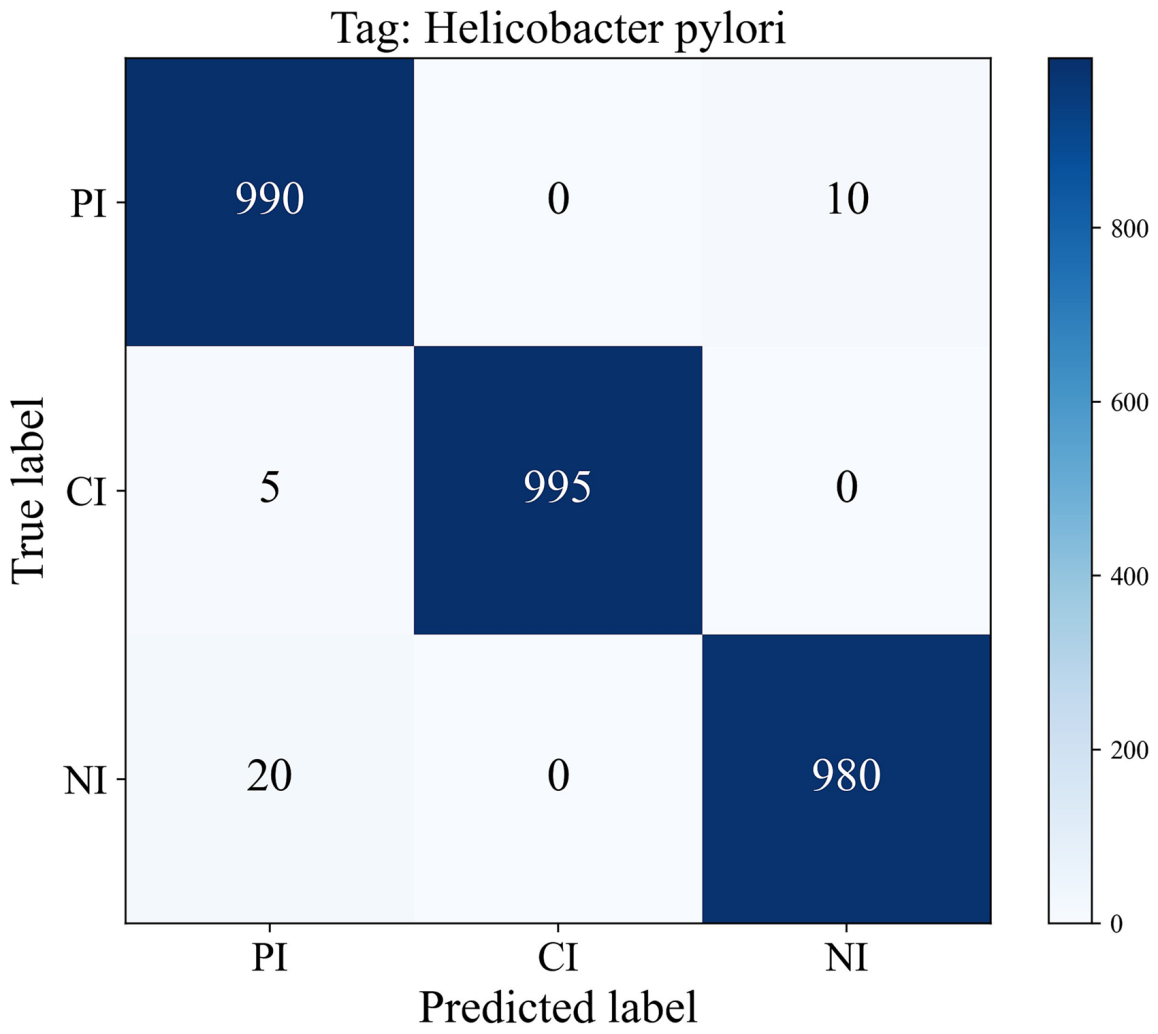
Confusion matrix of ten-fold cross-validation for HP identification.

In this section, we perform a more detailed analysis of the predictive accuracy and misclassification associated with the 1D-transformer model in different classification categories. As shown in Figure 3, the cumulative confusion matrix summarizes the results of the 10 cross-validations performed. The scheme demonstrates the consistent and accurate performance of the model in tumor staging and classification tasks. In the HP identification task, the model accurately diagnosed 990 cases as PI, 995 cases as CI, and 980 cases as NI.

Table 2 shows the results of the specificity, sensitivity and F1 score of the 1D-transformer model for the HP identification task. The results showed that the 1D-transformer performed stably in the HP recognition task, with a specificity above 98.8% for all categories, a sensitivity between 98.0 and 99.5%, and F1 scores between 98.3 and 99.7%. Notably, all three indicators in the NI category were at the highest level, and PI and CI also demonstrated consistent and reliable performance. Overall, the classification performance of the model for the HP subtasks was highly accurate and consistent.

**Table 2:** Specificity, sensitivity, and F1 scores of the 1D-transformer network model for different categories on the HP recognition task.

Classification	Category	Specificity	Sensitivity	F1_scores
HP identification	PI	98.8	99.0	98.3
	CI	100.0	99.5	99.7
	NI	99.5	98.0	98.5

In addition, we implemented 10-fold cross-validation to train and evaluate the 1D-transformer network model, resulting in 10 individual models. The performance of the HP recognition task is summarized in Table 3. The results show that most models achieved accuracy, specificity, sensitivity, and F1 score values in the range of 98.5 to 99.5%, indicating consistent recognition performance across different data partitions. A small number of models (e.g., Models 4 and 6) exhibited slightly lower performance; however, their metrics remained within 98.0–98.5%, with only marginal differences. These results demonstrate that, despite variations in data partitioning during cross-validation, the overall performance remains highly stable, reflecting the robustness and reliability of the proposed model for the HP identification task.

**Table 3:** Accuracy, specificity, sensitivity, and F1 score of the 1D-transformer network model for the HP recognition task.

Classification	Model	Accuracy	Specificity	Sensitivity	F1_scores
HP identification	Model 1	98.5	98.5	98.5	98.5
	Model 2	98.5	98.7	98.5	98.6
	Model 3	98.5	98.7	98.5	98.6
	Model 4	98.1	98.2	98.2	98.2
	Model 5	98.1	98.1	98.1	98.1
	Model 6	98.7	98.6	98.7	98.7
	Model 7	98.9	98.8	98.9	98.9
	Model 8	98.7	98.8	98.7	98.7
	Model 9	98.5	98.7	98.5	98.6
	Model 10	99.1	99.1	99.1	99.1

#### Overall evaluation results

**Figure 4:**
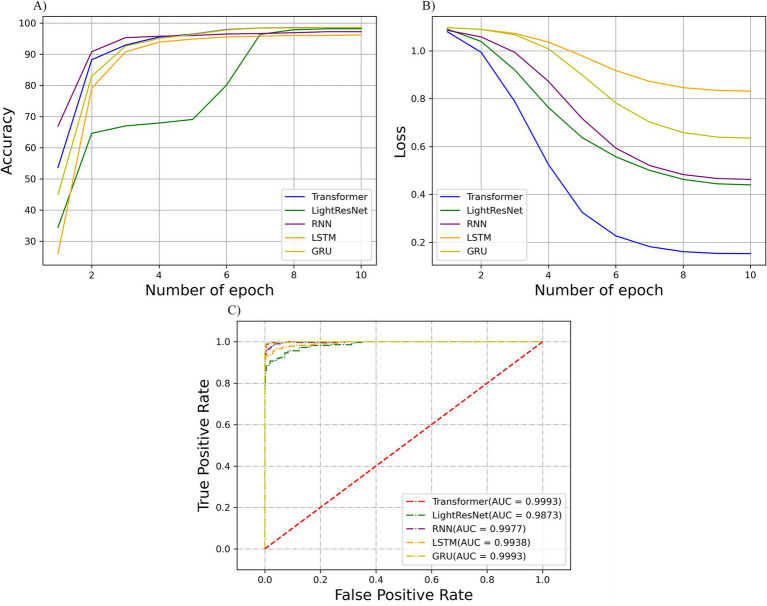
**(A)** Validation accuracy and **(B)** cross-entropy loss curves of the HP recognition task in CNN iterative training; **(C)** ROC curve of the test set and the corresponding AUC values.

Accuracy and cross-entropy loss are typically used as metrics when evaluating the performance and reliability of 1D-transformer models. As the learning iteration progresses, the accuracy and cross-entropy loss curves of the validation set gradually stabilize, indicating that there is no overfitting problem with the model. The accuracy curves and cross-entropy loss curves of different network models in the HP recognition task in this study are shown in Figures 4A,B, respectively. The higher performance and reliability of the 1D-transformer model over the other models were demonstrated through the accuracy and loss curves.

For comparison with other methods, considering the smaller data dimension, we chose LightResNet, BiRNN, BiLSTM, and BiGRU, as well as the XGBoost, SVM, and MLP machine learning algorithms as comparison models. LightResNet is an improved network model based on the ResNet architecture. By simplifying the network structure (retaining only one residual block and five core layers) and using 3 × 1 small convolutional kernels, it significantly reduces computational complexity while maintaining the advantage of residual learning and is suitable for the HP recognition task (13). A recurrent neural network (RNN) introduces a recurrent mechanism that enables the network to account for previous information while processing the current input. This feature makes RNNS perform well in tasks such as processing time series data and natural language processing (14). LSTM effectively addresses the long-term dependency of traditional RNNS by introducing input gates, forget gates, and output gates, enabling precise modeling of temporal dynamic features in HP data (15). GRU, as a simplified version of LSTM, combines the gating structure into update gates and reset gates, reducing the number of parameters and improving training efficiency, achieving a good balance of performance and complexity in the time series modeling of HP data (16). Multilayer perceptrons (MLPs) are a class of feedforward neural networks that can perform nonlinear mapping and classification of input features by stacking multiple fully connected layers and adding nonlinear activation functions between the layers. MLPs have good fitting ability for small- and medium-scale datasets and can learn complex relationships between signal features in HP classification tasks. On the other hand, the support vector machine (SVM) is a supervised learning model based on statistical learning theory and is widely used in classification tasks. XGBoost is an ensemble learning algorithm that gradually improves the accuracy of the model through a combination of additive models and decision trees. By evaluating these contrast models, we can fully analyze the performance of the 1D-transformer model in the HP classification recognition task.

Table 4 shows the performance metrics of our 1D-transformer, LightResNet, BiRNN, BiLSTM, BiGRU, SVM, and XGBoost algorithms on the HP recognition task, including accuracy, specificity, sensitivity, and F1 score. A comparative analysis revealed that our 1D-transformer model demonstrated superior performance in the HP recognition task, with the accuracy, specificity, sensitivity, and F1_scores produced by our model being 98.9 ± 0.28, 98.8 ± 0.14, 99.4 ± 0.27, and 98.9 ± 0.28, respectively. Furthermore, to quantitatively evaluate the classification performance of the 1D-transformer model, we plotted the receiver operating characteristic (ROC) curves of the HP recognition task under different network models and calculated the area under the curve (AUC) values. In terms of the transformer, LightResNet, RNN, LSTM, and GRU, the corresponding AUC values were 0.9993, 0.9873, 0.9977, 0.9938, and 0.9993, respectively (as shown in Figure 4C). These results further validate the superior performance of the 1D-transformer model for this task.

**Table 4:** Performance evaluation of the 1D-transformer, LightResNet, BiRNN, BiLSTM, BiGRU, SVM, and XGBoost algorithms in HP classification.

Algorithm types	Accuracy	Specificity	Sensitivity	F1_score
1D-transformer	98.9 ± 0.28	98.8 ± 0.14	99.4 ± 0.27	98.9 ± 0.28
LightResNet	98.2 ± 0.75	99.1 ± 0.37	98.2 ± 0.71	98.2 ± 0.74
1D-BiRNN	97.2 ± 0.53	98.6 ± 0.24	97.3 ± 0.54	97.2 ± 0.59
1D-BiLSTM	96.2 ± 0.58	98.1 ± 0.27	96.2 ± 0.57	96.1 ± 0.61
1D-BiGRU	97.3 ± 0.43	98.2 ± 0.21	97.4 ± 0.42	97.3 ± 0.44
XGBoost	95.7 ± 1.0	96.0 ± 0.5	95.6 ± 0.9	95.6 ± 0.9
SVM	82.5 ± 1.2	92.5 ± 1.2	82.9 ± 2.0	83.0 ± 1.8
MLP	88.1 ± 2.0	91.8 ± 0.9	88.5 ± 1.8	87.9 ± 1.4

### SHAP visualization analysis

This study reveals the role of the model’s features in HP category prediction through SHAP aggregated view analysis (Figure 4). The visualization results visually presented the relative importance of the 16 key features and their predictive trends: the positions of each data point on the *X*-axis reflected the SHAP values (positive for positive correlation, negative for negative correlation), and the color gradients represented the size of the feature values (red for high values, blue for low values). This two-dimensional presentation method clearly characterizes the differential contribution patterns of different features to the model’s prediction of HP categories.

The distribution of the contributions of 16 key features as the model predicts classification is shown in Figure 4. The SHAP values of different features differed significantly, among which features 14 (RAC), 5 (diffuse redness), 11 (map redness), 4 (mucous overlay), 3 (thickened folds), and 8 (xanxoma) were identified as key predictors. Specifically, as shown in Figure 4A, when the model predicted the PI categories, the high values of feature 11 and feature 8 were distributed mainly in the SHAP-positive region, indicating that significant map redness and xanthoma features increased the model’s ability to predict the PI categories. In contrast, the high values of features 14, 5, 4, and 3 were concentrated mainly in the negative area, suggesting that the RAC, diffuse redness, mucus coverage, and thickened folds manifestations weakened the model’s predictive confidence in the PI and made it more likely to be judged as other categories.

As shown in Figure 4B, when the model predicts the CI category, the high values of features 4 and 3 are concentrated mainly in the positive SHAP area, indicating that the enhancement of mucus coating, thickened folds, and dotted redness significantly increases the possibility of the model predicting the CI category. The high values of features 14 and 11 were distributed in the negative area, suggesting that the RAC and the red feature of the map actually weakened the confidence of the CI category. Notably, feature 5 (diffuse redness) is distributed in both positive and negative areas, suggesting that its role in CI discrimination has certain nonlinear characteristics and may be related to the complex interaction of local morphological changes.

As shown in Figure 4C, when predicting the NI category, unlike the previous two categories, the high values of feature 14 are concentrated mainly in the positive region, indicating that when the RAC features are enhanced, the model is more inclined to predict the CI. Moreover, the high values of features 5, 11, 3, 4, and 8 are generally distributed in the negative area, indicating that abnormal morphological features such as mucus attachment, chicken skin-like changes, vascular exposure, thickened folds, and punctate redness all reduce the model’s prediction probability for the NI category, thereby demonstrating that the model can effectively capture the significant differences between the infected and non-infected states.

**Figure 5:**
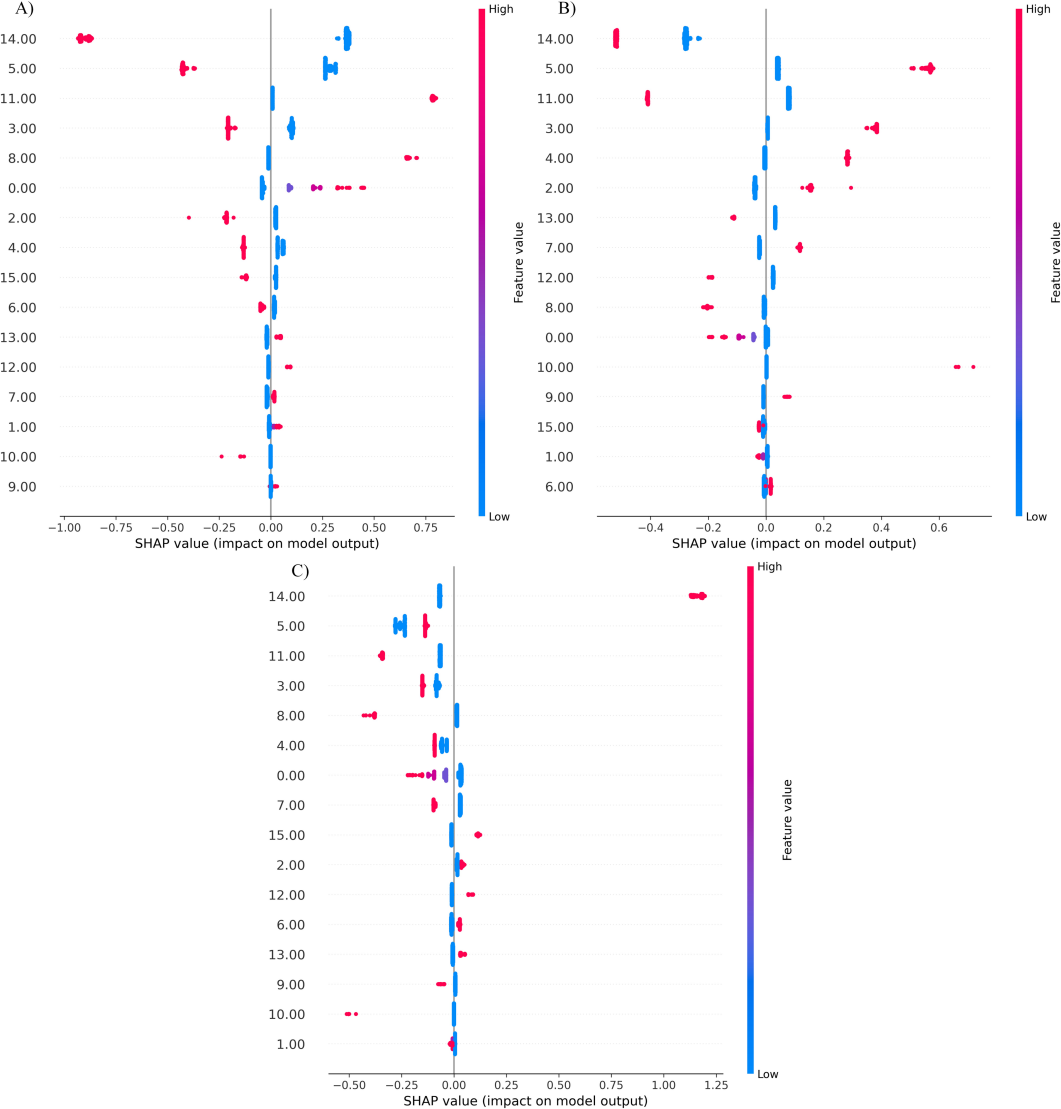
Distribution of SHAP feature contributions of the transformer model in the HP three-class classification task **(A–C)**. Each point represents the feature contribution of a single sample, with the horizontal coordinate being the SHAP value; red represents high feature values, and blue represents low feature values.

**Figure 6:**
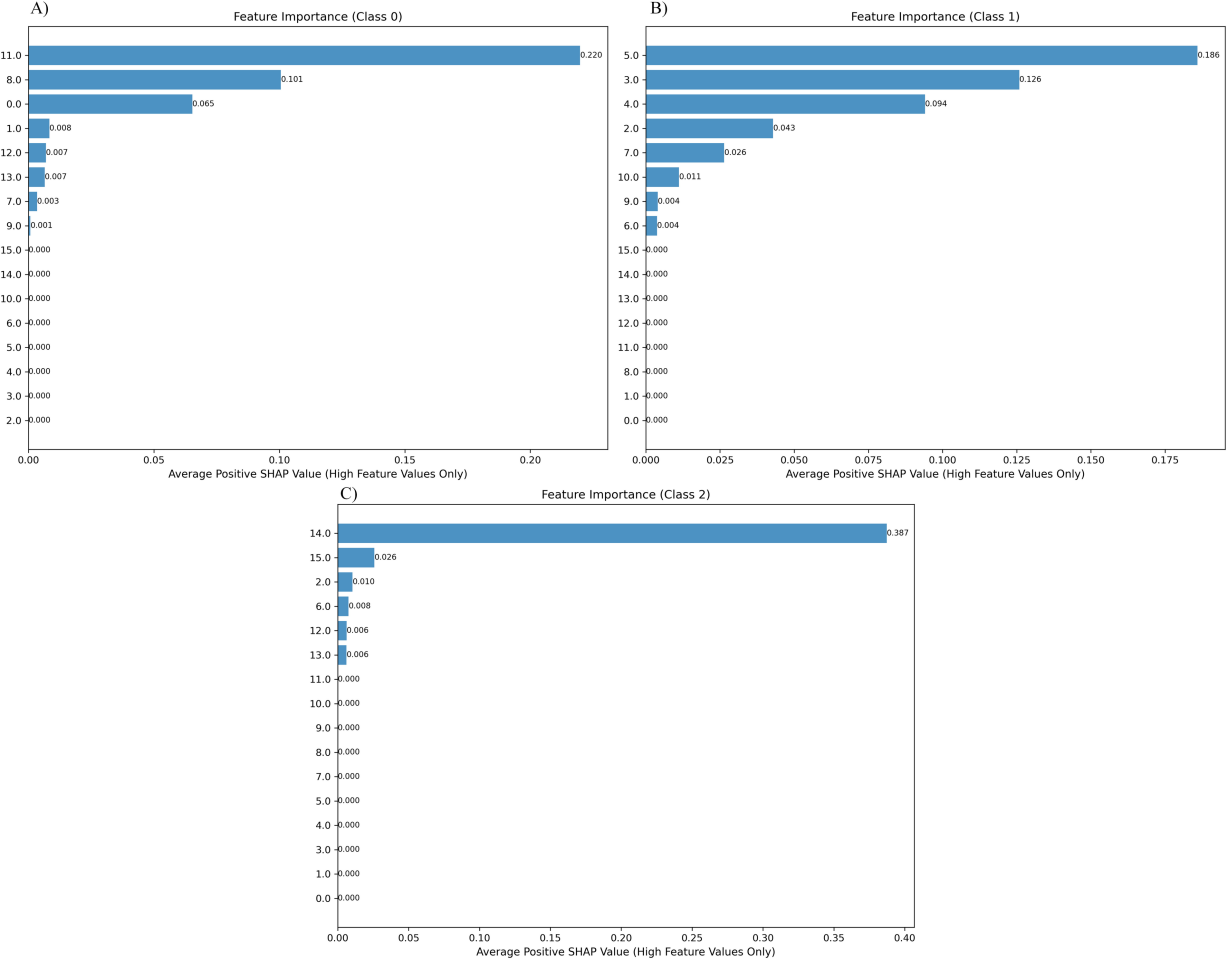
Feature contribution analysis results of the transformer model in the HP three-class classification task based on positive SHAP averages. **(A)** PI categories, **(B)** CI categories, **(C)** NI categories. The feature importance is sorted by SHAP values, and the matrix diagram visually shows the contribution of each covariate in the final prediction process.

The feature importance analysis results based on high eigenvalue positive SHAP contributions, corresponding to three types of predictions, namely, PI (Figure 5A), CI (Figure 5B), and NI (Figure 5C), are shown in Figure 5. Specifically, for each category of SHAP values, only the positive contribution portion corresponding to the high eigenvalue is retained (the rest is set to zero), and its average is calculated to highlight the key features that have a positive impact on the model’s predictions. In this study, the transformer model is used for classification, and the SHAP method is used to quantify the global contribution of each feature to the prediction results. By averaging the positive SHAP values of all samples, the top three most influential features for each category were screened out: In the PI category, feature 11 (0.220) contributed the most, indicating that redness in the map was most diagnostically significant for preexisting HP infection, followed by feature 8 (0.101), feature 0 (0.065), and feature 1 (0.008); in the CI category, feature 5 (0.186) contributed the most, indicating that diffuse redness was the most diagnostically significant for current HP infection, followed by feature 3 (0.126), feature 4 (0.094), and feature 2 (0.043); and in the NI category, feature 14 (0.387) dominated, indicating that the presence of RAC was most significant for diagnosing uninfected HP, followed by feature 15 (0.026). Overall, the results indicated that predictions in different categories relied on differentiated feature combinations, and the model was able to accurately identify and utilize these key features for effective classification, demonstrating strong feature recognition ability and stable decision logic (Figure 6).

## Discussion

*Helicobacter pylori* (HP) is classified as a Group 1 carcinogen by the World Health Organization and is closely associated with diseases such as chronic gastritis, peptic ulcer and gastric cancer. Its precise diagnosis and standardized management are at the core of clinical treatment. There are still many practical pain points in traditional detection methods. Pathological biopsy relies on the sampling experience of the operator, and rapid urease testing has a certain false negative rate because of factors such as bacterial colonization density (8, 9). Real-time judgment of the HP infection status during endoscopy can help endoscopists quickly understand the status of the gastric background mucosa to assist in subsequent diagnosis and treatment.

The types and characteristics of early gastric cancer vary across the three different backgrounds of *H. pylori* infection. For example, in the case of *H. pylori* infection, differentiated gastric cancer is more likely to occur in the context of gastric mucosal atrophy. Gastric cancer that is not infected with *H. pylori* is less common (16) and often presents as undifferentiated cancers such as signet ring cell carcinoma and fundus adenocarcinoma. Differentiated cancers, including those with characteristic endoscopic manifestations such as reddish depression (RD), are often found after radical *H. pylori* treatment. However, owing to histological changes in the surface structure and the various manifestations of RD, early gastric cancer is more insidious and difficult to distinguish from benign RD and is not easily detectable in the context of atrophy and intestinal metaplasia (17, 18). The distinction between the PI group and the NI group is not merely for the need of epidemiological classification, but rather to precisely correspond to two different clinical and pathological scenarios, thereby more clearly revealing: how the endoscopic characteristics and diagnostic difficulty of gastric cancer systematically change due to the different ‘soil’ of growth, that is, the infection background. This distinction has direct clinical significance for improving the precise screening and identification strategies for early gastric cancer.

The Kyoto Gastritis Classification and previous studies have shown that the main cause of atrophy and intestinal metaplasia is HP infection (19) because for the detection of atrophied or intestinal metaplastic gastric background mucosa, HP infection or previous infection status is considered first. However, in this study, atrophy or intestinal metaplasia still comprised 10.1% of the cases in the HP-negative group, and atrophy and intestinal metaplasia did not significantly contribute to the characteristics of the model. For patients with chronic atrophic gastritis or intestinal metaplasia who had no history of active HP infection or had not received antibacterial treatment, the cause was likely the unintentional eradication of *H. pylori* in the treatment of other infectious diseases or the spontaneous disappearance of *H. pylori* (20). Therefore, diagnosing HP status solely on the basis of a history of infection or eradication is not accurate. Most of the current research on AI-assisted diagnosis of HP infection focuses on the binary differentiation between “current infection” and “no infection,” but in clinical practice, the “previous infection” population is often confused with the other two states because of possible residual inflammatory traces or nonspecific changes after repair of the gastric mucosa (13, 14, 21). This study, for the first time, uses AI technology to achieve three-dimensional typing of HP infection, which can identify the three infection states and assist in clinical decision-making more accurately.

This study aims to achieve efficient identification and key feature extraction of HP infection status through deep learning models to improve the accuracy and automation of clinical diagnosis. We systematically compared five mainstream deep learning models (transformer, LightResNet, BiRNN, BiLSTM, and BiGRU) with three classical machine learning algorithms (SVM, XGBoost, and MLP), and the results revealed that the transformer model achieved the best performance in terms of classification accuracy (98.9 ± 0.28%), specificity (98.8 ± 0.14%), sensitivity (99.4 ± 0.27%), and F1 score (98.9 ± 0.28%). The stability and generalization ability of the transformer model were also significantly better than those of the other models. Compared with the local receptive field feature extraction of CNN-like models and the sequence-dependent modeling of RNN-like models, the transformer, which relies on its self-attention mechanism, has stronger global feature modeling and nonlinear representation capabilities and can capture richer structural semantic information in low-dimensional feature spaces. It has shown significant advantages in the task of recognizing the complex shapes of HP organizations.

In the model interpretability study, the contribution of each clinical feature to the prediction of high-risk population identification was systematically evaluated using the SHAP value analysis method. By constructing a global bee colony map and a bar chart based on high eigenvalue positive SHAP contributions, we visually demonstrated the importance rankings of different features in model decision-making and the directions of their influence. Map redness (SHAP value of 0.220), xantheloma (SHAP value of 0.101), atrophy (SHAP value of 0.065), and intestinal metaplasia (SHAP value of 0.008) were key features for identifying the PI. Diffuse redness (SHAP 0.186), thickened folds (SHAP 0.126), mucus coverage (SHAP 0.094), and nodular changes (SHAP 0.043) are key features for identifying CI. The presence of RAC (SHAP 0.387) and ridge redness (SHAP 0.026) are key features for identifying NI. This finding is similar to those of previous studies (22–25). The classification of gastritis in the Kyoto scoring system revealed that redness on the map is a characteristic manifestation of PI, diffuse redness is a characteristic manifestation of CI, and RAC is a characteristic manifestation of NI, all of which are consistent with the results of this study. However, a single feature is not a criterion for determining the status of HP infection. In clinical practice, it should be evaluated on the basis of a combination of various features and endoscopic results. Therefore, the advantages of artificial intelligence can be more prominent at this time, and a comprehensive judgment can be made in combination with the manifestations of various features.

This study has several limitations that need to be improved upon in subsequent work. In terms of sample selection, the samples in this study were derived from a single-center population, which may introduce selection bias. The clinical characteristics of single-center samples are relatively concentrated, making it difficult to fully cover HP infection patients in different regions and different disease spectra, which may affect the generalizability of the AI model. External validation with multiple centers and multiple sample sizes is still needed. Although the imaging features of this study were discussed by two experienced doctors to ensure consistency to the greatest extent possible, the method could still not completely eliminate the influence of subjective judgment and lacked an absolute objective gold standard for calibration. In addition, the study relied on artificially designed features, which may have failed to fully exploit the deep information in the data. Furthermore, regarding the definition of “previous *H. pylori* infection,” we rely on histopathological evidence combined with retrospective treatment records. This reliance on treatment history may introduce recall bias and incomplete record issues, resulting in inaccurate information about the infection status. The fact that other methods such as serological tests or breath tests were not routinely combined in all cases is a limitation of this study. Future prospective research designs can integrate multiple mode of detection to further improve the accuracy of infection status classification.

## Conclusion

This study applies a deep learning network model to classify HP infection status and can accurately diagnose current, past and uninfected HP status, providing a feasible and efficient new approach in this field. Compared with the other models, the transformer model demonstrated superior performance and reliability in the three-class classification task of HP infection, with higher accuracy, specificity, sensitivity and F1 scores. In addition, the SHAP value analysis revealed that map redness was a key manifestation for identifying the PI, diffuse redness was a key feature for identifying the CI, and RAC was a key feature for identifying the NI.

## Data Availability

The raw data supporting the conclusions of this article will be made available by the authors, without undue reservation.
